# Quadruple and quintuple transpapillary stenting without predilation for complex hilar biliary obstruction using a novel 7-Fr ultratapered plastic stent

**DOI:** 10.1055/a-2678-9739

**Published:** 2025-08-20

**Authors:** Takehiko Koga, Makoto Fukuyama, Yusuke Ishida, Naoaki Tsuchiya, Keisuke Matsumoto, Yi-Ling Ko, Fumihito Hirai

**Affiliations:** 1 38208 Department of Gastroenterology and Medicine, Faculty of Medicine, Fukuoka University Hospital, Fukuoka, Japan

Management of hilar biliary obstruction (HBO) often requires transpapillary multistenting; however, the insertion of more than three stents into severe hilar biliary strictures remains technically challenging, even after predilation 1 . Recently, a novel 7-Fr ultratapered plastic stent (Crane stent, SB-KAWASUMI, Kanagawa, Japan) has shown high insertability in endoscopic ultrasound-guided drainage procedures 2 3 4 5 . Given their structural advantages, we used ultratapered stents for transpapillary multistenting in two challenging HBO cases ( Video 1 ).

Quadruple and quintuple transpapillary stenting is successfully performed without predilation using the novel 7-Fr ultratapered plastic stents.Video 1

**Patient 1** was an 81-year-old woman with hepatocellular carcinoma who developed HBO due to immune checkpoint inhibitor-induced sclerosing cholangitis. During the initial endoscopic retrograde cholangiopancreatography (ERCP), three 7-Fr plastic stents were placed in segments B2, B7, and B8 ( Fig. 1 ); however, the patient subsequently developed segmental cholangitis in B3, necessitating reintervention. First, the previously placed stent in B2 was removed, and a 7-Fr plastic stent was placed in B3. The ultratapered stent was then selected as the fourth stent and was successfully inserted into B2 ( Fig. 2 ). No dilation device was used at any stage of the procedure. Postoperatively, the patient’s cholangitis improved.

**Fig. 1:**
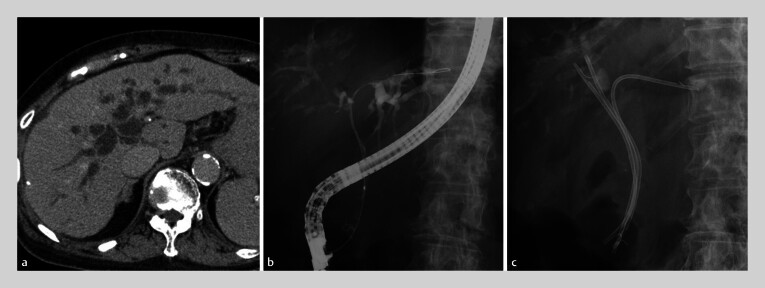
Initial imaging of patient 1 showing: **a** on a computed tomography image, hilar biliary obstruction and intrahepatic bile duct dilatation; **b, c** on fluoroscopic images during the initial endoscopic retrograde cholangiopancreatography, three 7-Fr plastic stents that were placed in B2, B7, and B8.

**Fig. 2:**
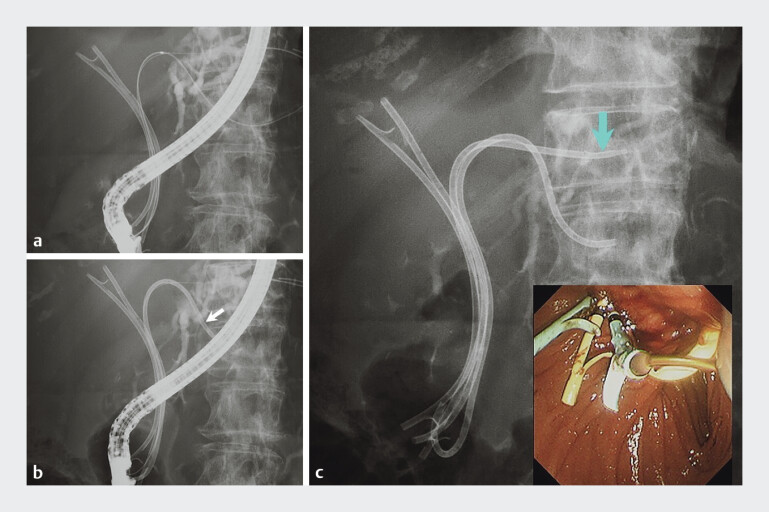
Images during the reintervention performed in patient 1 showing: **a** removal of the previously placed stent in B2, with guidewires inserted into B2 and B3; **b** a 7-Fr plastic stent placed in B3 (white arrow); **c** a 7-Fr ultratapered plastic stent that was successfully placed in B2 as the fourth stent (blue arrow), with no dilation device used during the procedure (inset, endoscopic view).

**Patient 2** was a 69-year-old man with unresectable gallbladder cancer who presented with HBO. Magnetic resonance cholangiopancreatography revealed isolation of tertiary bile duct branches. During the initial ERCP, 7-Fr plastic stents were placed in B3 and B8 ( Fig. 3 ); however, his jaundice persisted, necessitating reintervention. After the previously placed stents had been removed, three 7-Fr plastic stents were placed in B2, B3, and B5. Subsequently, the ultratapered stents were selected as the fourth and fifth stents and were successfully inserted into B4 and B8 ( Fig. 4 ). No dilation device was used at any stage of the procedure. The patient’s jaundice improved, enabling the subsequent initiation of chemotherapy.

**Fig. 3:**
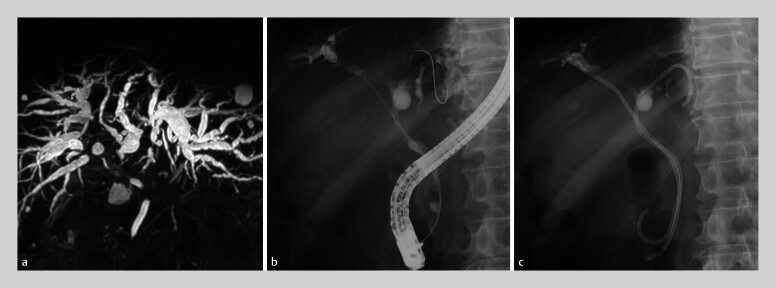
Initial imaging of patient 2 showing: **a** on magnetic resonance cholangiopancreatography image, intrahepatic bile duct dilatation and isolated tertiary bile duct branches; **b, c** on fluoroscopic images during the initial endoscopic retrograde cholangiopancreatography, two 7-Fr plastic stents that were placed in B3 and B8.

**Fig. 4:**
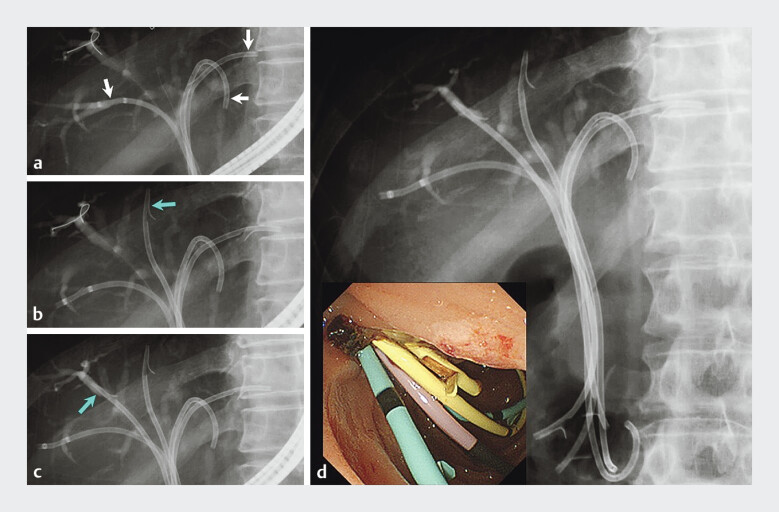
Images during the reintervention performed in patient 2 showing: **a** three 7-Fr plastic stents that were placed in B2, B3, and B5 (white arrows) after removal of all the previously placed stents; **b** a 7-Fr ultratapered plastic stent that was successfully placed in B4 as the fourth stent (blue arrow); **c** a further 7-Fr ultratapered plastic stent that was subsequently placed in B8 (blue arrow) as a fifth stent, with no dilation device used during the procedure (inset, endoscopic view); **d** post-procedural fluoroscopic and endoscopic images.

In both cases, more than three stents were successfully placed without predilation. The seamless transition between the guidewire, inner sheath, and stent tip in the ultratapered plastic stent system contributes to its excellent insertability ( Fig. 5 ). This structural design may offer significant advantages for complex multistenting in cases of HBO.

**Fig. 5:**
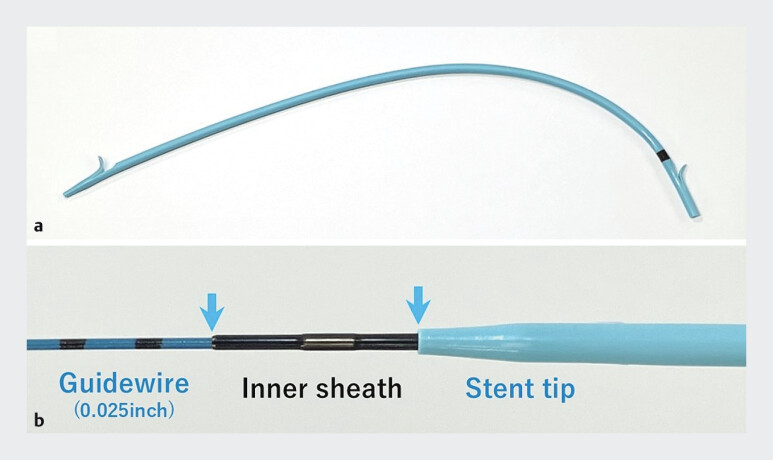
Photographs of: **a** the novel 7-Fr ultratapered plastic stent (Crane stent, SB-KAWASUMI, Kanagawa, Japan); **b** the tip of the stent system, which is compatible with a 0.025-inch guidewire and features an ultratapered structure, giving a seamless transition between the guidewire, inner sheath, and stent tip, and leading to excellent insertability.

Endoscopy_UCTN_Code_TTT_1AR_2AZ

## Notes

Endoscopy E-Videos https://eref.thieme.de/e-videos
*E-Videos* is an open access online section of the journal *Endoscopy* , reporting on interesting cases and new techniques in gastroenterological endoscopy. All papers include a high-quality video and are published with a Creative Commons CC-BY license. Endoscopy E-Videos qualify for HINARI discounts and waivers and eligibility is automatically checked during the submission process. We grant 100% waivers to articles whose corresponding authors are based in Group A countries and 50% waivers to those who are based in Group B countries as classified by Research4Life (see: https://www.research4life.org/access/eligibility/ ). This section has its own submission website at https://mc.manuscriptcentral.com/e-videos .
